# Probabilistic working memory representations in human cortex guide behavior

**DOI:** 10.1101/2025.11.17.688881

**Published:** 2025-11-17

**Authors:** Ying Zhou, Clayton E. Curtis, Daryl Fougnie, Kartik K. Sreenivasan

**Affiliations:** 1 Division of Science and Mathematics, New York University Abu Dhabi; 2 Department of Psychology and Center for Neural Science, New York University; 3 Center for Brain and Health, New York University Abu Dhabi

**Keywords:** working memory, probabilistic population coding, fMRI, uncertainty, decoding, memory-guided decisions, Bayesian

## Abstract

Models of working memory make fundamentally different commitments to the architecture of individual memories. Information-sparse models conceptualize individual memories as single point estimates agnostic to meta-cognitive variables such as uncertainty. In contrast, information-rich models propose memories are encoded as probability distributions over feature space that embed memory uncertainty in the shape of the distribution. To distinguish these accounts, we constructed probability distributions of memory from participant’s iterative reports of motion direction on each trial. Remarkably, the idiosyncratic shape of these distributions (e.g., asymmetry) on single trials matched the shape of neural probability distributions decoded from fMRI patterns measured from occipital and parietal cortex. Consistent with information-rich models, the neural representation of an individual memory encodes more than the memorized feature; its variance (i.e., width and asymmetry) encodes idiosyncrasies whose read-out predicts memory behavior.

## Introduction

We know surprisingly little about the precise format by which working memory representations are stored in the brain. For example, when we temporarily maintain a recently encountered direction of random dot motion in working memory, do we store a probability distribution over the space of all possible motion directions, or do we instead represent a specific direction of motion? The former view is inspired by probabilistic models that propose the activity of populations of neurons encodes a full probability distribution over feature space^1–3^. We refer to these models as *information rich*, because the same population of neurons encodes memory content and memory uncertainty in a multiplexed representational space akin to a probability distribution over possible feature values^4–6^. In contrast, high-threshold^7,8^, mixture^9,10^, and continuous attractor models^11,12^ are *information sparse*, because they represent memories as a single point estimate in feature space, with memory uncertainty potentially encoded in a separate neural population. Information sparse representations preclude a direct readout of uncertainty from the population storing the memory. In standard attractor models of working memory^12^, for instance, a memory is encoded by the increased response gain, or *bump,* in units tuned to the memorized feature. While memory errors stem from the magnitude and direction of the bump’s drift over time^11^, critically, no information about memory uncertainty can be discerned from the population activity. Ultimately, these two classes of models make fundamentally distinct claims about the format of memory representations, the mechanisms by which individual memories are transformed into behavioral output, and the origins of meta-cognitive information, such as uncertainty. Thus, adjudicating between them is essential for understanding the neural basis of working memory.

**Figure 1.**
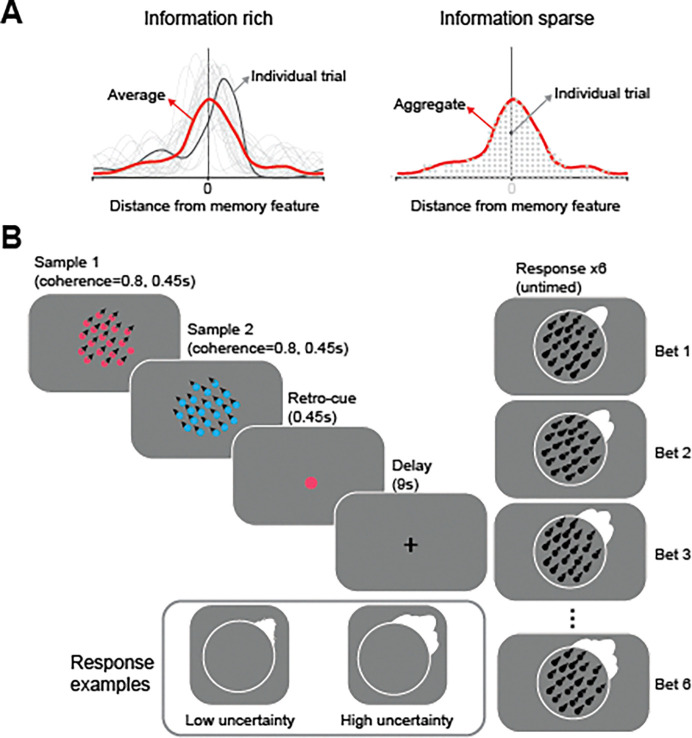
**(A)** Aggregated versus trial-wise representations. Left: when individual memories are full probability distributions (each trial is indicated by a gray line), as predicted by information-rich models, the trial-averaged distribution (indicated by the red line) is roughly symmetric and centered on the memory feature. Right: when individual memories are point estimates in feature space (each trial is indicated by a gray dot), as predicted by information-sparse models, the trial-averaged distribution (indicated by the red line) is identical to the average distribution shown in the left panel. Thus, information-rich and information-sparse models make indistinguishable predictions when data is aggregated over trials. **(B)** Trial schematic of the betting game task. Participants were presented with two dot-motion stimuli followed by a retro-cue, and retained the cued motion direction over a memory delay period. They reported the cued motion direction by placing six distributions around the probed circle to create an average distribution representing their memory on that trial. The inset depicts example responses when participants had low or high uncertainty about their memory. A video of the task is available at https://osf.io/zc3w4/.

Unfortunately, the ability to elucidate the architecture of individual memories is limited by experimental approaches that aggregate data over trials, which result in identical predictions for information-rich and -sparse models. Imagine a scenario in which the same feature value is remembered on several trials. Information-rich models would represent those memories as full probability distributions with roughly the same mean, but with distribution shapes that vary across trials. Trial-wise idiosyncrasies in the shape of the distribution, such as its asymmetry, may skew beliefs about the feature value in memory^13–15^. However, this information would be lost when averaging over trials, resulting in a roughly symmetric distribution centered on the memorized feature value (Figure 1A, left). Critically, the same distribution can result from aggregating point estimates generated by information-sparse models (Figure 1A, right), making it impossible to distinguish the two models. Thus, ignoring trial-wise idiosyncrasies can obscure information-rich representations and prevent us from fully understanding how the brain encodes memory (for related discussions, see^16,17^).

In the current study, we tested the hypothesis that our memories are influenced by how uncertainty varies asymmetrically across the feature space of an encoded memory. This hypothesis predicts that the specific shape of neural probability distributions that encode individual working memories should predict idiosyncrasies of memory-guided behavior. To test this prediction, we derived estimates of neural probability distributions of individual memories using a Bayesian decoder^18,19^ along with a recently-developed procedure that allows participants to build probability distributions of their memory through sequential reports^13,14^ (Figure 1B). To preview, we found that both behavioral and neural probability estimates were highly idiosyncratic with asymmetric distributions over feature space that diverged markedly from symmetric distributions derived from averaging over trials. Critically, in several brain areas in occipital and parietal cortex, the shapes of the decoded neural distributions closely matched those of behavioral probability distributions on single trials. These results support information-rich models in which working memory content and uncertainty are encoded jointly within a single neural population as a complex probability distribution that sculpts memory-guided behavior.

## Results

### Decoding results

**Figure 2.**
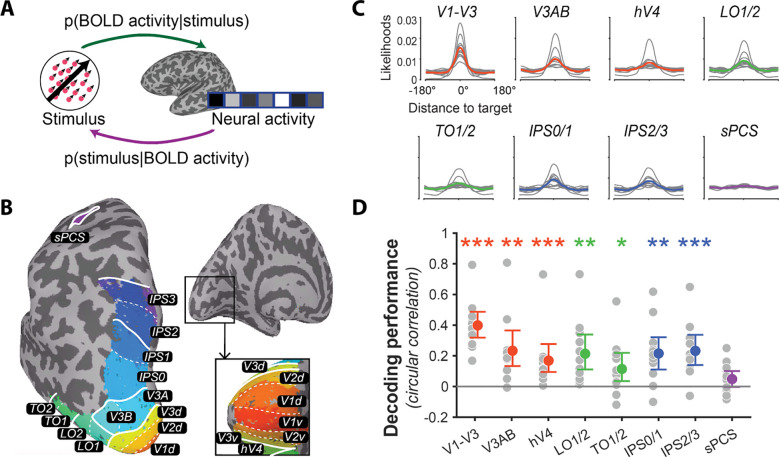
Decoding working memory representations. **(A)** Schematic of Bayesian decoding. TAFKAP first generates a model to predict BOLD activity given the stimulus (p(BOLD activity|stimulus); green arrow) and then infers a full posterior distribution for the stimulus given the BOLD activity (p(stimulus|BOLD activity); purple arrow). **(B)** Topographically organized regions of interest (ROIs) shown for the left hemisphere of a representative participant. Colors and labels indicate ROIs defined using a probabilistic atlas of retinotopy 27, with boundaries between ROIs indicated by white lines. ROIs sharing a confluent fovea were grouped together ^28,29^. **(C)** Decoded posterior distributions averaged over trials for each ROI. Individual trial distributions were aligned to a common reference of 0° and averaged over trials. Gray lines depict the centered posterior averaged across all trials for individual participants; colored lines are the group average. Color indicates anatomical grouping of ROIs: red for early visual regions, green for lateral occipito-temporal cortex, blue for parietal cortex, and purple for frontal cortex. **(D)** Circular correlation between the decoded and target motion directions. Gray dots represent individual participants, and colored dots indicate the group mean for each ROI. Stars indicate a significant difference between the actual decoding performance and a null simulation where the circular correlation was calculated with target directions randomly permuted across trials. For all figures, error bars indicate the 95% bootstrap confidence interval and stars denote significance (***: p < .001; **: p < .01; * p < .05, false discovery rate-corrected across ROIs with q=.05 where appropriate).

We collected fMRI data while participants performed a working memory task in which they were presented with two patches with moving dots and, after a memory delay, were asked to report the global motion direction of the cued patch (see Figure 1B and Experiment design for details). To identify individual memory representations encoded in the brain, we utilized a Bayesian decoder^20^ to derive a posterior probability distribution over the full motion space (0°–360°) from fMRI signals recorded during the delay period of individual trials (see Figure 2A and Bayesian decoding for details). Decoding was conducted separately for regions of interest (ROIs; Figure 2B) in retinotopically organized regions in visual cortex (V1-V3, V3AB, hV4), lateral occipitotemporal areas (TO1/2, LO1/2), posterior parietal cortex (IPS0/1, IPS2/3), and lateral frontal cortex (sPCS).

First, to compare our findings to previous work, we employed a standard approach that aggregates decoding results across trials. In each ROI, aligned the decoded distributions by setting the target to 0° and averaged the aligned distributions across trials (Figure 2C). On each trial, we used the circular mean of the decoded probability distribution to represent the decoded motion direction^19,20^. Consistent with previous studies using similar decoding methods^19,21–26^, we were able to decode memory content from occipital and parietal ROIs. To quantify decoding performance, we calculated the circular correlation between the mean of the decoded distribution and target motion direction on individual trials. This correlation was significantly higher than a null distribution generated by randomly permuting the target direction across trials. This finding was consistent in all ROIs except sPCS (all correlations ≥ .11; *p*s ≤ .018, Cohen’s *d*s ≥ .66; Figure 2D and Table S1).

**Figure 3.**
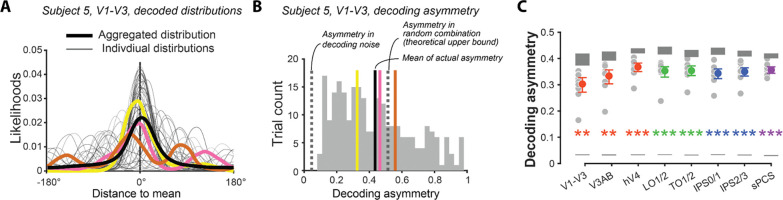
Asymmetry in decoded distributions. **(A)** Averaged versus individual decoded distributions for one sample participant and ROI. The thick black line indicates the posterior distribution averaged across trials, while the gray lines denote the decoded posterior distribution on individual trials. Posterior distributions for three example trials are highlighted in yellow, pink, and brown. **(B)** Asymmetry across trials for the same participant and ROI as in A. The light gray dashed line near 0 represents the asymmetry expected due to estimation noise alone (see decoding performance for details). The dark gray dashed line is the theoretical upper bound of asymmetry. The colored lines correspond to the example trials highlighted in panel A. **(C)** Asymmetry across ROIs. Gray dots represent individual participants and colored dots are the mean of the measured asymmetry. Light gray bars (bottom) indicate the amount of expected asymmetry from estimation noise, and dark gray bars (top) indicate the theoretical upper bound. The heights of bars indicate 95% bootstrap confidence intervals. Stars denote significant difference between measured asymmetry and asymmetry attributable to estimation noise.

The shapes of averaged decoded posterior distributions were not, however, representative of single-trial decoded distributions. In particular, individual trial posteriors often exhibited a marked asymmetry that was lost when the distributions were averaged across trials. As shown for an example participant and ROI in Figure 3, the shapes of individual trial posteriors were highly variable (Figure 3A) and exhibited a range of asymmetry that diverged from the trial-averaged level (Figure 3B). Of course, some degree of trial-wise asymmetry is expected - even if the underlying neural representation is perfectly symmetrical - due to noise inherent in the decoding process. This noise is inherent because the voxel tuning curves used to compute likelihoods in the posterior distributions are estimates, not ground truth. To determine if the asymmetries we observed could be attributed to estimation noise, we compared the asymmetries in the decoded data to those obtained from simulations of randomly re-centered decoded distributions. This re-centering procedure mimics the outcome of decoding with completely uninformative tuning curves by shuffling the likelihoods for all possible values, thereby capturing the maximal asymmetry expected from estimation noise alone. We found that the observed asymmetries in the decoded distributions were significantly larger than the asymmetries in the simulations (*p*s ≤ .001, Cohen’s *d*s ≥ 2.80; Figure 3C), confirming that they cannot be explained by estimation noise. Moreover, the observed asymmetries approached the theoretical upper bound estimated by randomly combining the left and right halves of posteriors from different trials, suggesting that the decoded representations are intrinsically and highly asymmetric (Figure 3C).

Next, we tested if the asymmetries in neural distributions are meaningful by asking whether they can predict asymmetries in memory-guided behavior. To do so, however, we required a behavioral task sensitive to the potential probabilistic nature of memory. Single reports about one’s memory are insufficient for this purpose. Thus, we employed our recently developed behavioral paradigm that uses multiple reports about a single memory item to estimate the distribution-level information contained in individual working memories^13,14^.

### Betting game response distribution

**Figure 4.**
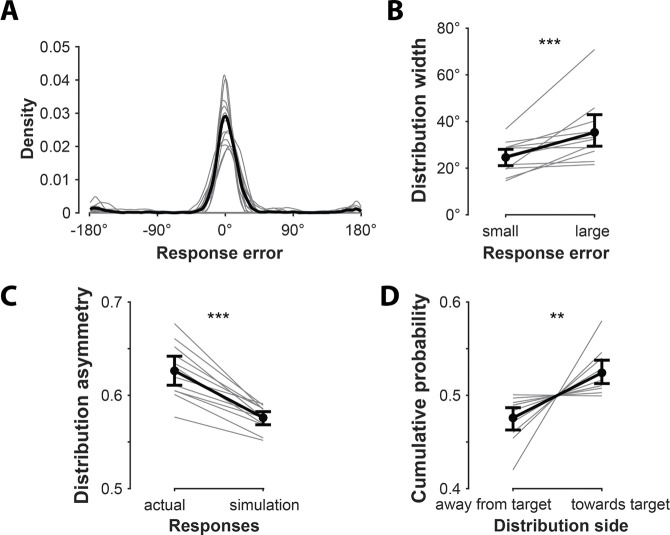
Behavioral performance. **(A)** The distribution of errors on participants’ first response. In this and following panels, gray lines indicate individual participants and the black line represents the average across participants. **(B)** Distribution width in trials with small vs. large error on the first response. The bet distribution was wider in trials with less accurate first responses, consistent with the notion that participants were indicating their memory uncertainty with the placement of their bets. **(C)** Distribution asymmetry (the cumulative probability on the larger side when the bet distribution was centered relative to the first response). The actual behavioral asymmetry of bet distributions was larger than that in simulated bet distributions where the signs of bets 2–6 relative to the first response were randomly changed. **(D)** Target information in distribution asymmetry. Cumulative probability was greater toward the target side, relative to the first response, than the opposite side. The stars in each figure indicate a significant difference between the two conditions.

On each trial, participants constructed a distribution reflecting their memory by placing 6 bets about target motion direction instead of a single report (see Figure 1B and Experiment design for details). To compare performance with traditional single-report tasks, we calculated the mean absolute error of the first response (Figure 4A). Error was lower for the first response (M = 22.49°, SEM = 2.70°) than subsequent responses (M = 25.26°, SEM = 2.60°; *p* < .001, Cohen’s *d* = 1.776), suggesting that the first response was an effortful best estimate of target identity. Moreover, the error on the first response was comparable to that reported in prior single-report studies of working memory for motion direction^30^, suggesting that multiple responses did not lead to strategy shifts on the first response relative to single-response paradigms ^13^.

A key question is whether bets 2–6 provide additional information about the target beyond the first bet, despite the fact that performance was individually worse for bets 2–6. For example, these extra bets may convey useful information about memory uncertainty; if participants’ memory about the motion direction is precise, they are likely to cluster their bets, but if not, they are likely to disperse their bets more widely (see inset in Figure 1B). Consistent with this, we found that trials with larger response errors were associated with broader bet dispersion (*p* < .005, Cohen’s *d* = .83; Figure 4B; see also^19^).

Reminiscent of the neural decoded distributions, bet distributions were asymmetric around the first bet. That is, when centered on the first response, the cumulative probability was larger on one side than the other. This asymmetry was significantly larger than that expected due to random response noise, as estimated by simulations in which the directions of subsequent responses were randomly flipped relative to the first response on each trial (see Behavioral performance for details; M = 0.63 vs. 0.58; *p* < .001, Cohen’s *d* = 2.32; Figure 4C). Critically, this asymmetry contained meaningful information about the target: the cumulative probability on the target side (M = 0.52) was significantly larger than on the opposite side (M = 0.48; *p* = .006, Cohen’s *d* = 1.07; Figure 4D). This asymmetry results in the mean of the bet distribution being a more faithful representation of the target direction (M = 18.90°, SEM = 1.77°) than the first bet (*p* = .001, Cohen’s *d* = .999). These results demonstrate that the asymmetric shape of trial-wise response distributions, arising from participants’ subsequent bets, conveys information about individual working memory targets. Notably, this information would be lost and participants’ knowledge about their memories would be underestimated if only the first response were collected, demonstrating the utility of multiple response paradigms.

### Correspondence between brain and behavior

**Figure 5.**
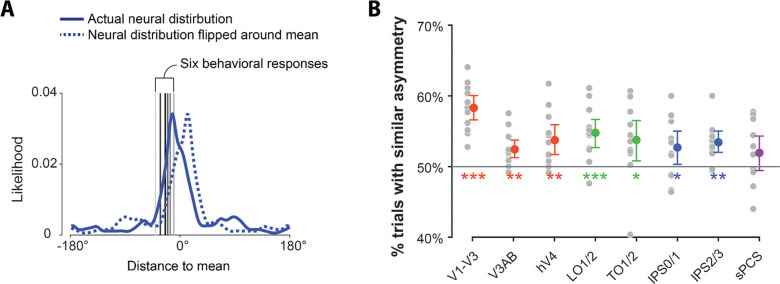
Brain-behavior correspondence. **(A)** Schematic of the asymmetry comparison between neural and behavioral estimates. Filled blue lines represent the actual neural distribution centered at its mean for an individual trial, and dashed blue lines depict the neural distribution reflected about its mean. Both distributions share the same mean and width, differing only in their asymmetry. Vertical gray lines from darker to lighter indicate behavioral responses 1–6. We compared the sum of likelihoods at the six behavioral responses for the actual and flipped neural distributions on individual trials. **(B)** Brain-behavior correspondence was quantified as the percentage of trials on which the actual neural distribution fit the behavioral response better than the flipped neural distribution. Gray dots denote the correspondence for individual participants, and colored dots denote the mean of correspondence for all participants in different ROIs. Stars indicate that the actual neural distribution fits the behavioral responses better than the flipped neural distribution.

In order to establish that the shape of our neural and behavioral distributions represents meaningful information about individual memories, it is essential to demonstrate a correspondence between neural and behavioral estimates at the single-trial level. We first replicated previous findings^19^ that the mean and width of neural estimates can predict behavioral reports (Figure S1). However, this analysis ignores the full distribution shape, and therefore is insufficient to adjudicate between information-rich and -sparse models. To test correspondence between the shapes of our neural and behavioral estimates beyond the mean and width, we examined whether the neural distribution predicted the placement of bets on individual trials better than the same distribution reflected about its mean (Figure 5A). Importantly, flipping the neural estimate in this manner results in a distribution with the same mean and width as the original, but with reversed asymmetry. Information-rich models predict that the original neural estimate would correspond with behavior better than the flipped estimate, while information-sparse models predict no difference. Supporting information-rich models, we found that the sum of the likelihoods over the six behavioral responses in the neural distribution was significantly larger than that in the distribution flipped around its mean in all ROIs except sPCS (*p*s ≤ .010, Cohen’s *d*s ≥ .829; Figure 5B, Figure S2). These results indicate that idiosyncrasies of probabilistic neural estimates, such as the asymmetry, in addition to their mean and width, can predict characteristics of memory-guided reports.

### Causes of asymmetries in individual memories

#### What causes asymmetries in memory?

**Figure 6.**
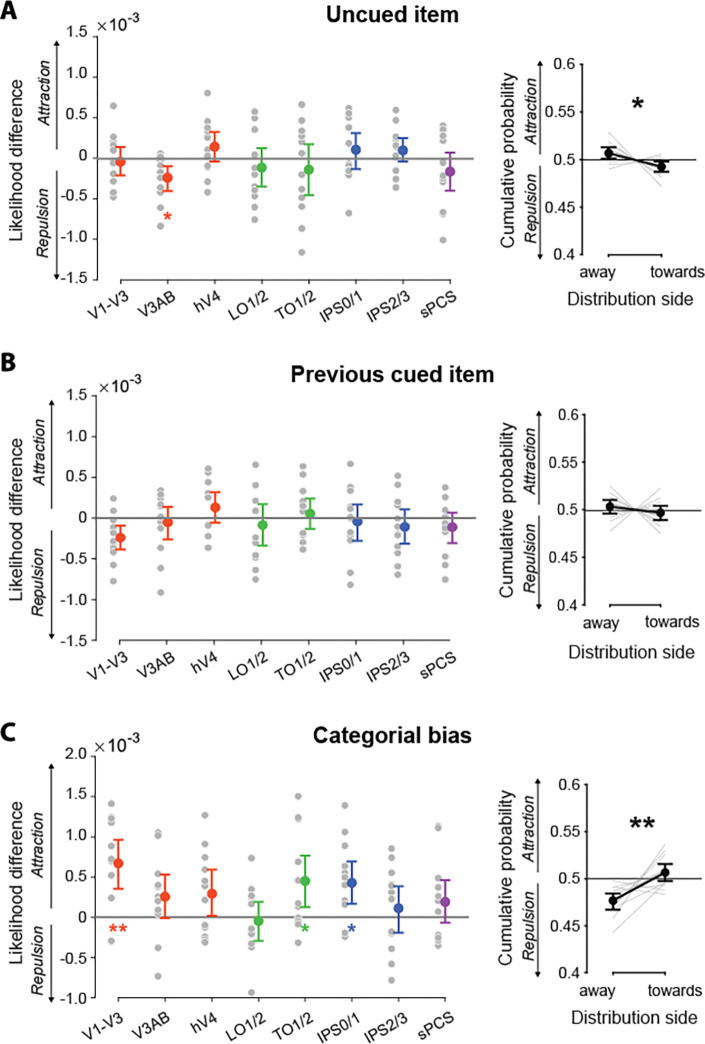
Causes of asymmetries in neural and behavioral distributions. **Left column**: Neural bias, quantified by calculating the likelihood over the motion direction of interest and subtracting the likelihood over the same position in the neural distribution reflected about its mean. Positive (negative) values indicate an attractive (repulsive) bias, respectively. Gray dots represent individual participants, colored dots indicate the group mean for each ROI, and stars indicate bias that differed significantly from zero. **Right column**: Behavioral bias, quantified as the difference between the cumulative probability on the side of the distribution away from vs. toward the motion direction of the interested cause, relative to the first response. Gray lines represent individual participants, the group mean is shown in black, and stars indicate significant difference between the distribution sizes on the two sides **(A)** Bias caused by the uncued motion direction on the same trial. Left: There was a significant repulsive bias away from the uncued direction in V3AB. Right: There was a significant repulsive bias from the uncued direction in the behavioral distributions. **(B)** Bias caused by the cued direction on the previous trial. Left: A trend towards repulsive bias in V1-V3 did not survive FDR correction. Right: Despite a trend towards a repulsive bias (7 of 12 participants), no significant bias was observed in the behavioral distributions. **(C)** Categorial bias caused by nearby canonical directions. Left: There was a significant repulsive bias towards the nearby canonical directions in V1-V3, TO1/2, and IPS0/1. Right: There was a significant attractive bias toward the nearby canonical directions in the behavioral distributions.

We evaluated two potential sources that were predictable across trials, while acknowledging that other sources such as non-Gaussian noise^6,10^ may also contribute to memory asymmetries but are substantially more difficult to quantify. First, there is ample evidence that memories can be biased by information that was previously observed but is currently task-irrelevant. This includes uncued items from the current trial^31,32^ as well as previously relevant memory items from the preceding trial^33–35^. To ascertain whether task-irrelevant items contributed to the asymmetries we observed, we measured whether they systematically biased the neural and behavioral distributions. In V3AB, the likelihood in the decoded probability distribution over the uncued motion direction was significantly smaller than the likelihood over the same position in the distribution flipped around the mean (*p* = .004, Cohen’s *d =* .860; Figure 6A, left). There was a corresponding reduction in the size of the behavioral distribution toward, compared to away from, the uncued motion relative to the first bet (*p* = .023, Cohen’s *d =* .674; Figure 6A, right). Together, these results are consistent with the idea that inter-item competition from uncued memory items creates a repulsive bias in memory representations^36^ and suggest that this bias contributed to our observed asymmetries in neural and behavioral probability distributions. When we repeated the same analysis for the cued motion direction from the previous trial, we observed a similar repulsive bias in the decoded distribution in V1-V3; however, this effect did not survive multiple comparisons (*p* = .064, Cohen’s *d =* .803). No bias was observed for the other ROIs (*p*s ≥ .107, Cohen’s *d*s ≤ .360) or the behavioral (*p* = .200, Cohen’s *d =* .237) distributions (Figure 6B), perhaps due to the long interval between trials^37,38^.

Another factor that could contribute to asymmetries in memory is a systematic categorical bias toward canonical directions (vertical, horizontal, and oblique)^39,40^. Supporting this idea, we found that decoded probability distributions in V1-V3 (*p* = .001, Cohen’s *d =* 1.170), TO1/2 (*p* = .015, Cohen’s *d =* .752), and IPS0/1 (*p* = .006, Cohen’s *d =* .807) had a greater likelihood over nearby canonical directions than on the opposite side relative to the mean. The bet distribution was similarly larger on the side facing the nearby canonical directions than on the opposite side relative to the first bet (*p* < .001, Cohen’s *d =* 1.080; Figure 6C and Figure S3). Thus, attraction to canonical feature values may also contribute to memory asymmetry. The asymmetry we observed is not just limited to the elements tested here, but is likely the result of a complex interplay of factors, such as trial-wise variability^41^.

## Discussion

Here, we tested the hypothesis that asymmetrical uncertainty across mnemonic feature space shapes neural probability distributions, which in turn predicts idiosyncrasies in memory-guided behavior. Our findings support this hypothesis and demonstrate that memory and uncertainty are jointly represented across the visual hierarchy and that the architecture of individual memories is complex, probabilistic in nature, and drives memory-guided behavior. These results represent a mutual validation of single-trial behavioral and neural probabilistic memory estimates, and support information-rich models of working memory positing that multiplexed information about memory and uncertainty is encoded in the same neural populations. One way to store multiplexed information in neural populations is proposed by the theory of probabilistic population codes: the brain knows how patterns of neural activity relate to visual features and uses population activity to estimate both the likely feature and the uncertainty that comes with it^1,3,42–44^. In contrast, information-sparse theories conceive of memories as point estimates in feature space that lack notions of uncertainty. Some information-sparse models, such as continuous attractors^11,12,45,46^, can in principle incorporate information about memory uncertainty; in such cases, our findings place major constraints on precisely how uncertainty is represented. For example, models where uncertainty is encoded separately from memory content but influences the representation through feedback mechanisms ^47,48^ are inconsistent with our observations as any information about asymmetry would be lost and could not influence behavior. Put another way, asymmetric uncertainty suggests that memory and uncertainty are jointly encoded.

Furthermore, our framework of trial-wise probabilistic brain-behavior comparison provides a critical tool for differentiating the contributions of distinct brain regions to working memory. While memory features can be simultaneously decoded from multiple brain regions including visual, parietal, and prefrontal cortices^19,49–51^ (see^52,53^ for reviews), a precise understanding of each region’s role remains elusive^54–56^. Importantly, it is misleading to infer that these regions encode information of the same quality based on single estimates (e.g., mean or width) of memory; even if the neural probability distributions estimated from these areas have identical mean and width, differences in their shape could signify meaningful differences in the information that they represent. Our approach, which identifies nuanced but behaviorally relevant aspects of the shape of individual memory representations, offers a powerful means for distinguishing the quality of working memory representations in different brain regions, and is thereby able to clarify their unique functional roles. Our results suggest that early visual areas, often considered less important for working memory^55,57,58^, contain sufficient information to guide complex memory-guided behaviors involving uncertainty and bias.

The field’s reliance on simple reports and aggregate analyses is partly based on the assumption that such methods adequately characterize useful working memory properties. For example, average response error across set sizes has been used to infer working memory capacity limits^9,59,60^. However, there are several problems with this perspective. First, our results argue that the enterprise of characterizing an average working memory state may miss the point; memory states were highly variable across trials, with few resembling the aggregate (Figure 3A). Rather than illuminating a purported qualitative average memory state, aggregate approaches may mask the underlying mechanisms that produce working memory representations^61^. Second, there is no established theory explaining how uncertainty in neural memory representations is converted into behavioral reports, despite being an essential inferential step. One might assume that participants take an optimal sample from memory, such as the mean of a memory distribution. However, we found that averaging multiple behavioral reports was more accurate than individual reports, suggesting that each individual sample is suboptimal. Behavioral responses may instead reflect noisy or partial samples from the underlying probabilistic representation^13,62,63^. Alternatively, the underlying distributions may be too complex for one report to summarize^6^. These results underscore the need for a theory of how neural uncertainty in memory is converted into behavioral reports (an area typically relegated to decision-making^64^), in order to make meaningful advances in linking brain and behavior.

Our work highlights several key considerations for future research. First, examining working memory representation on a per-trial basis is essential, as this approach can reveal additional information, such as trial-specific uncertainty, that is lost in aggregated analyses. Second, on individual trials, relying on a single point estimate - either a summary of neural decoding or a summary of behavioral report - is insufficient; instead, estimating the full probability distribution can provide a more comprehensive understanding of working memory representation and memory-guided behavior. Finally, tracking brain-behavior relationships at the single-trial level is crucial for deepening our understanding of working memory^16,65,66^. By adhering to these principles, we constrain working memory models by highlighting that successful theories must account for the joint encoding of both mnemonic and metacognitive information. Further, we show that deviations from the aggregate have consequences – they make successful predictions about what participants will report and with how much certainty they report it.

## Methods

### Participants

All procedures were approved by the New York University Abu Dhabi (NYUAD) Institutional Review Board. Based on sample sizes from previous fMRI studies using Bayesian decoding^18,19,30,67^, we recruited fifteen neurologically healthy volunteers (5 males, 27.5 ± 4.5 years old) from the university community. All reported normal or corrected-to-normal visual acuity and normal color vision. Two participants were excluded from subsequent analysis because their response error exceeded two standard deviations above the group mean, and one withdrew from the study before completing data collection. The remaining 12 participants (5 males, 28.7 ± 4.1 years old) completed two (2 participants) or three (10 participants) MRI sessions. Participants were compensated for their time with AED 120 (~USD 33) per session, plus a performance-based bonus of up to AED 50 (~USD 13).

### Experimental design

We generated stimuli and interfaced with the MRI scanner, button box, and response dial using MATLAB software (The MathWorks, Inc., Natick, MA) and Psychophysics Toolbox 3 (Brainard, 1997). Stimuli were presented using a PROPixx DLP LED projector (VPixx Technologies, Inc., Saint-Bruno, QC, Canada) located outside the scanner room and projected through a waveguide and onto a translucent screen located at the head of the scanner bore. Participants viewed the screen at a viewing distance of 88 cm through a mirror mounted on the head coil. A scanner trigger synchronized stimulus presentation and image acquisition.

Each trial (Figure 1B) began with two sequential colored motion patterns (80% coherence; radius = 4 degrees of visual angle [dva]) presented for 450 ms each, separated by a 200 ms interval. Each motion pattern comprised 70 moving dots (0.16 dva) traveling at 9 dva/s, with motion directions drawn, without replacement, from nine evenly spaced categories (20°–360°), jittered ±5° to prevent participants from forming categorical representations of the motion. Participants memorized both motion directions for later recall. After 200 ms, a 450ms color retrocue (0.2 dva) indicated which motion direction to retain. Following a 9000 ms delay, participants adjusted a black probe motion pattern (100% coherence) using an MR-compatible dial (Current Design, Inc., Philadelphia, PA) to match the cued direction and confirmed their response with a button press.

Instead of a single response, as in typical continuous report working memory tasks, participants made 6 bets to report the target motion direction on each trial^13,14^. Each bet was displayed as a von Mises distribution (circular SD = 10°) that moved on the screen as participants adjusted the dial, and the sum of the six bets formed the final response distribution for that trial. Participants could spread the bets freely, with the cumulative distribution updated in real time to reflect all bets placed so far plus the new bet reflected by the current dial position. A video of the task is available at https://osf.io/zc3w4/.

To encourage accurate reporting of something resembling internal uncertainty, points were awarded based on the height of the final response distribution over the target motion direction:

Points=Heightoffinaldistributionattargetdirection*500.

The first bet was assigned double the height of bets 2–6 (i.e., worth twice as many points) to encourage accuracy on the first bet. To discourage participants from only stacking bets, we implemented diminishing rewards for stacking. Specifically, if the center of the new bet is at b, the existing cumulative response distribution is y, and v is the von Mises distribution representing the new bet, then the new cumulative response distribution y’ after the new bet is:

$$y^{\prime }=y+v\times \left(1\right.-\;\left.{{Height}_{\mathrm{at}\;b}}^{0.4}\right),where\;0.4\;is\;the\;penalty\;parameter.$$

Thus, the higher the existing cumulative distribution at the new bet’s location, the smaller the incremental gain in height after adding that bet. This penalty was visually represented in real time within the response display. Consequently, to maximize their points (and thus bonus payment), participants were incentivized to cluster bets when confident about the target motion direction and to spread them when uncertain (see Figure 1B, inset).

After participants placed all six bets, a 1500 ms feedback screen displayed the final response distribution, the target direction, the points earned on that current trial, and the cumulative total. The inter-trial interval was 10500 ms. Each scanning session consisted of 9 or 10 runs of 9 trials.

### fMRI Methods

#### MRI data acquisition.

Scanning sessions were conducted at the NYUAD Brain Imaging Core on a Siemens Prisma (3T) MRI scanner with a 64-channel head/neck coil. Functional BOLD scans were acquired using an EPI pulse sequence with 44 slices and a voxel size of 2.5 mm isotropic (4x simultaneous-multi-slice acceleration; FoV = 200 × 200 mm, no in-plane acceleration, TE/TR = 30/750 ms, flip angle = 50°, bandwidth = 2604 Hz/pixel, 0.51 ms echo spacing, P → A phase encoding). To correct for local spatial distortions, we estimated a field map of the field inhomogeneities by acquiring pairs of spin echo images with normal and reversed phase-encoding directions with an identical slice prescription to the functional data and no simultaneous-multi-slice acceleration (TE/TR = 36/3100ms, 3 volumes per phase encoding direction). To enable precise localization of functional data, we collected T1-weighted whole-brain anatomical scans using a MP-RAGE sequence with 208 slices and a voxel size of 0.8 mm isotropic (FoV = 256 × 240 mm, TE/TR = 3.45/2400 ms, flip angle = 8°, bandwidth = 220 Hz/Pixel). Slice positioning and FoV were individually adjusted to ensure full-brain coverage. To further improve the surface extraction, we acquired additional 3D T2-weighted SPACE sequences in the same resolution as the T1-weighted anatomical scan (FoV = 256 × 240 mm, TE/TR = 564/3200 ms, bandwidth = 744 Hz/Pixel).

#### MRI data preprocessing.

Our preprocessing followed the Minimal Preprocessing Pipeline of the Human Connectome Project (MPP)(Glasser et al. 2013), which we optimized for use on NYUAD’s High Performance Computing cluster to run multiple participants in parallel. Briefly, the structural MRI scans (T1 and T2) were aligned, brain extracted, and corrected for readout distortions before being projected into MNI space using Boundary Based Cross Modal Registration (BBR), and then finally bias-field corrected using the combination of T1 and T2 contrasts. Functional data was corrected for using MPP’s *fMRIVolume* pipeline. After correcting scanner-specific gradient distortions, the BOLD time series underwent motion correction through rigid body registration to align with the single-band reference gradient echo EPI volume. Susceptibility artifacts were corrected utilizing the pair of Spin Echo EPI volumes with opposite phase encodings (AP and PA directions). Subsequently, BBR was used to co-register the single-band reference gradient echo EPI volume with the corrected T1w volume. This registration transformation was then consolidated into a single transformation step that was applied to the BOLD series. Since the volumetric processing of the MPP pipeline only produces corrected functional scans in MNI space, we used FSL’s *applywarp* algorithm to resample the BOLD data into native space. We visually inspected the overlap of the native BOLD data with the native corrected T1w and T2w volumes to ensure the accuracy of the registration. All analyses were conducted in corrected native volume space. Finally, we removed linear trends from the BOLD series and normalized (z-score) across all time points within each run.

#### Regions of interest (ROIs).

We used FreeSurfer (http://surfer.nmr.mgh.harvard.edu/) to construct brain surfaces from the corrected T1w anatomical scans using the *recon-all* command, and identified the following participant-specific ROIs in surface space from the probabilistic map of visual topography^27^ using the *atlas* command in Neuropythy^68^: hV4, V1, V2, V3, V3A, V3B in occipital visual cortex; LO1, LO2, TO1, TO2 in lateral occipito-temporal cortex; IPS0, IPS1, IPS2, IPS3 in parietal cortex; and sPCS in frontal cortex. We combined ROIs that share the same foveal confluence^28,29^ (V1, V2, and V3; V3A and V3B; IPS0 and IPS1; IPS2 and IPS3; LO1 and LO2; TO1 and TO2; Figure 2B). Finally, these ROIs were projected into native volume space.

### Bayesian decoding

In order to decode the motion direction stored in working memory on each trial, we used the averaged z-scored BOLD response for each voxel from 6.75s to 9s after the onset of the delay period as the input to a Bayesian decoder, TAFKAP^20^. The output of TAFKAP is a posterior probability distribution of each stimulus value over the whole feature space. Specifically, it first uses testing data to create a generative model to predict the neural activity given a stimulus value, then infers the posterior of stimulus value given the neural activity by applying Bayes’ theorem on unlabeled testing data (see the illustration in Figure 2A).

In the generative model, the voxel response given the stimulus (i.e., the target motion direction) was modeled as a multivariate normal distribution. Specifically, the average response of each voxel $b_{i}$ was determined by its tuning function $f_{i}(s)$ plus a random noise $\varepsilon_{i}$:

$$b_{i}=f_{i}(s)+\varepsilon_{i}.$$

The tuning function $f_{i}(s)$ of each voxel i was approximated by a weighted sum of nine basis functions $g_{k}(s)$ evenly distributed across the feature space:

$$\begin{array}{l} f_{i}\left(s\right)=\sum_{k}W_{ik}g_{k}\left(s\right),\\ g_{k}\left(s\right)={\left(0,\cos\left(\frac{\pi }{90}\left(s-\phi_{k}\right)\right)\right)}^{5}, \end{array}$$

where $g_{k}(s)$ is the k-th basis function, which peaks at direction $\phi_{k}$, and $W_{ik}$ is a weighting matrix that determines the weights of each basis function $g_{k}(s)$ for each voxel i. The noise $\varepsilon_{i}$ for each voxel is assumed to be correlated between voxels, with covariance Ω, such that ε∼N(0,Ω) and $b_{i}∼N\left(f_{i}(s),\Omega \right)$. The probability of a multivoxel activity pattern $b={\left[b_{i}\right]}^{T}$ is therefore given by:

$$p\left(s;W,\Omega \right)=N\left(f\left(s\right),\Omega \right),$$

where $f(s)=[f_{i}(s)]$ are the values of each voxel’s tuning functions given stimulus s, and voxel weight W and covariance structure Ω are the free parameters needed to be estimated.

The free parameters were estimated from the data in a leave-one-run-out cross-validation procedure. That is, each participant’s dataset was divided into training and testing samples, such that each run served as the testing sample once. The training samples were used to estimate the free parameters in the generative model. Specifically, the voxel weight W was estimated by

$$\overset{ˆ}{W}=BG^{T}{\left(GG^{T}\right)}^{-1},$$

where $B=[b_{\mathrm{train}}^{\left(t\right)}]$ is a matrix of multivoxel activity in all training samples and $G=[g(s_{\mathrm{train}}^{\left(t\right)})]$ is a matrix of the values for the nine basis functions given stimulus s in the training samples, and t indexes trials. The covariance matrix Ω can be theoretically estimated by a sum of noise independent between voxels and noise shared among all voxels,

$$\Omega =\left(1-\rho \right)diag\left(\tau^{2}\right)+\rho \tau \tau^{T}+\sigma^{2}WW^{T},$$

where $\tau^{2}={\left[\tau_{i}^{2}\right]}^{T}$ are the variance parameters of noise that is independent between voxels, and ρ is a global correlation parameter for noise that is shared among all voxels; $\sigma^{2}$ is a variance parameter for noise that is shared between voxels with similar tuning properties; and $WW^{T}$ indicates the similarity between tuning curves. In addition to this theoretical covariance matrix, the model also considered the empirical sample variance, which can be calculated by

$$\Omega_{\mathrm{sample}\;}=\frac{1}{N_{\mathrm{train}\;}}(B-\overset{ˆ}{W}G)(B-\overset{ˆ}{W}G{)}^{T}.$$

Considering both the theoretical and empirical covariance, the new covariance matrix $\Omega_{\mathrm{new}}$ was modeled as the sample covariance matrix “shrunk” to the theoretical covariance matrix Ω. The degree of shrinkage is determined by a free parameter λ,

$$\Omega_{\mathrm{new}\;}=\lambda \Omega +\left(1-\lambda \right)\Omega_{\mathrm{sample}\;}.$$

The parameters of this covariance model (τ,ρ,σ and λ) were estimated by numerically maximizing their likelihood under the training samples, conditioned on the estimate of W.

After estimating the free parameters using the training samples, we inferred the posterior of stimulus value given the multivoxel responses in each testing trial by applying the Bayes’ theorem:

$$p\left(b_{test};W,\Omega \right)=\frac{p\left(b_{test}|s;W,\Omega \right)p(s)}{\int \;p\left(b_{test}|s;W,\Omega \right)p(s)ds},$$

where a flat prior p(s) reflecting the equal possibility of the nine stimulus categories from which the target values were sampled in the experiment, and the normalization constant in the denominator was computed numerically over the whole feature space. We used a discrete approximation of the posterior, evaluating $p\left(b_{\mathrm{test}};W,\Omega \right)$ at 180 equally spaced directions in the whole feature space (0°–360°).

To account for the uncertainty of model parameters, TAFKAP uses a bootstrap aggregating method. We resampled the trials in the training data with replacement multiple times to generate many resampled training datasets. Each resampled training dataset had the same number of trials as the training samples. For each resampled training dataset j, a set of free parameters $W_{j}$ and $\Omega_{j}$ was estimated using the method mentioned above. The posterior distribution of stimulus given the multivoxel response in each testing trial in iteration j of bootstrapping was then computed as

$$p\left(b_{test};W_{j^{\prime }},\Omega_{j}\right)=\frac{p(b_{test}|s;W_{j^{\prime }},\Omega_{j})p(s)}{\int p(b_{test}|s;W_{j^{\prime }},\Omega_{j})p(s)ds}.$$

For each testing trial, this decoding was performed multiple times based on the parameters estimated using each resampled training dataset. The final decoding result of each testing trial was the averaged posterior distributions across all the bootstrapping iterations

$$p_{\mathrm{bag}\;}(b)=\frac{1}{N_{\mathrm{bootstrap}\;}}\sum_{j}p\left(s|b;W_{j^{\prime }}\Omega_{j}\right).$$

The number of bootstrapping iteration ($N_{\mathrm{bootstrap}}$) was determined by a stopping criterion based on Jensen-Shannon divergence. In our testing process, if the largest Jensen-Shannon Divergence between the new and the last 100 posterior distribution samples was smaller than 1e-8, or the bootstrapping iterations were larger than 5 × 10^4^, we terminated the bootstrapping process.

We smoothed the final posterior distribution with a sliding window of 10° to facilitate comparisons with the bet distributions, which were summations of von Mises distributions with a circular standard deviation of 10°.

### Decoding performance

For each trial, we defined the decoded motion direction as the circular mean of the posterior distribution obtained from the Bayesian decoder^19,20^. We assessed decoding performance using the circular correlation between the decoded and the actual motion directions^19,20^. For each participant and ROI, we computed the circular correlation and compared it to a null distribution obtained by randomly permuting the target direction over trials and then recomputing the circular correlation 1000 times. We report the *p*-values at the individual participant level as the proportion of null circular correlations larger than the actual circular correlation in Table S1. For group-level inference for the circular correlation results, we used a nonparametric permutation-based test^26^ because there was no *a priori* reason to believe that the data would be normally distributed. We compared the *t-*statistic derived from the group *t*-test of the actual circular correlation against 0 to a null distribution of *t-*statistics obtained by comparing the null correlations for all participants in each permutation with 0. The *p-*value on group level was the proportion of null *t*-statistics larger than the actual *t*-statistic, which are reported in Figure 2D. The effect size is reported as Cohen’s d^69^.

The asymmetry of the decoded posterior distribution around its mean was quantified by the dissimilarity caused by flipping the distribution. If a distribution is asymmetric around its mean, flipping it around the mean would result in a distribution dissimilar to the original distribution; in contrast, flipping a symmetric distribution around the mean would result in an identical distribution. We computed the overlap between the original and flipped distributions to measure the similarity between them. This measure has the advantage of not requiring any distributional assumptions such as symmetry, unimodality, and well-established parametric forms, and has been shown to be able to considerably improve the interpretability of data analysis results in psychological research^70^. The overlap η between distribution $f_{A}(x)$ and distribution $f_{B}(x)$ was determined by

$$\eta (A,B)=\sum min\left[f_{a}(x),f_{b}(x)\right],$$

which is larger for more similar distributions^71^.

To confirm that the asymmetry in our decoded posterior distributions was not simply caused by the noise in estimating voxels’ tuning curves, we compared the actual asymmetry with simulations where voxels’ turning curves were random noise without any meaningful information. For each participant and ROI, the simulation was generated by re-centering the posterior distributions at randomly permuted locations and taking the mean of all these re-centered distributions. The re-centered distributions were used to quantify the asymmetry of the simulated distribution as described above for the actual posterior distributions. This process was repeated 1000 times to derive a null distribution of asymmetry. To compare the actual asymmetry with that caused by estimation noise and get the statistical results reported in Figure 3C, we used the nonparametric permutation-based test described above to compare the actual *t*-statistic derived from comparing the actual asymmetry for all participants with 0 to the null distribution of *t-*statistics obtained by comparing the asymmetry of estimation noise for all participants with 0 in each permutation. The *p*-value at the group level was the proportion of null *t*-statistics larger than the actual *t*-statistic.

To further contextualize our asymmetry measure, we compared the actual asymmetry with the theoretical upper bound of asymmetry, which was simulated by randomly combining the left side of the distribution on each individual trial with the right side of the distribution on another trial when all distributions were centered at their mean, and recomputing the dissimilarity caused by flipping. This process was repeated 1000 times for each individual trial and the average was taken as the theoretical upper bound asymmetry for each individual trial. The theoretical upper bound asymmetry for each participant and ROI was the average of all individual trials.

### Behavioral performance

We quantified behavioral response error as the circular distance between the first response and the target stimulus as in previous studies using single response paradigms (Figure 4A). To demonstrate that the subsequent responses on individual trials incorporates information about memory uncertainty, we compared the width of bet distribution (the absolute value of the difference between the minimum and maximum bet error on a given trial) in accurate and inaccurate trials (Figure 4B). Trials were labeled accurate or inaccurate for each participant based on a median split of the error of the first response. The group-level statistical comparison of bet distribution width between accurate and inaccurate trials was calculated using a nonparametric permutation-based test. We first performed a paired *t*-test across participants between accurate and inaccurate trials. The resulting *t*-statistic was compared against a null distribution generated by randomly permuting the trial labels (accurate vs. inaccurate) within each participant and recomputing the *t-*statistics 1000 times. The group level *p*-value was the proportion of null *t*-statistics larger than the observed *t*-statistic.

To quantify the asymmetry of the bet distribution, we used the cumulative probability on the larger side of the bet distribution after centering it on the first response (Figure 4C). To confirm that the asymmetry created by subsequent responses was not simply an artifact of response noise (i.e., that randomly placed later bets could, by chance, produce apparent asymmetry around the first response), we compared it with a simulation where the direction of subsequent responses relative to the first response on individual trials was randomly switched. The simulation process was repeated 1000 times for each participant to yield a null distribution of asymmetry levels. We report group-level statistics from the nonparametric permutation-based test comparing the actual and simulated asymmetry levels, as described above.

To confirm the asymmetry of the bet distribution contained meaningful information about the target direction, we compared the cumulative probability on the side containing the target with that on the other side when the bet distribution was centered relative to the first response (Figure 4D). Group-level statistics were computed using a nonparametric permutation-based test comparing the cumulative probability on the target and non-target sides, as described above.

### Brain-behavior correspondence

We first tested brain-behavior correspondence in the mean and width of the distribution estimates by computing binned-correlation between the mean and width of behavioral and neural estimates as in^19^. For each participant, trials were sorted into four bins according to decoding error. The behavioral response error was computed by averaging across trials within each bin. We then pooled data points across participants (four data points per participant - one per bin) after removing the mean of each participant. Pearson correlation between the neural and behavioral estimates was then computed based on the pooled data (Figure S1). We compared the correlation coefficients to the null distribution obtained by permuting the data points in the pooled dataset to obtain a *p*-value. For visualization, we added the mean of each participant back to the data when plotting the binned-correlations. We repeated the same binning analysis using uncertainty instead of error.

To confirm the brain-behavior correspondence in the shape of the distribution estimates, we examined whether the asymmetry of the neural decoded distribution could predict behavioral responses. Specifically, we tested whether the actual neural decoded distribution fits the behavioral responses better than the same neural distribution flipped around its mean. For each individual trial, the likelihood of getting the actual 6 behavioral responses based on the neural distribution was calculated by

$${\text{Likelihood}\;}^{i=6}\prod_{i=1}^{i=1}P\left({\mathrm{Reponse}}_{i}\right)∼log\left(\prod_{i=1}^{i=6}P\left({\mathrm{Reponse}}_{i}\right)\right)=\sum_{i=1}^{i=6}log\left(P\left({\mathrm{Reponse}}_{i}\right)\right)$$

where P is the likelihood of ${reponse}_{i}$ at the neural distribution. If the likelihood of the actual decoded distribution is higher than that of the flipped decoded distribution in an individual trial, this trial was labeled as a matching trial. The brain-behavior correspondence for each ROI and individual participant was calculated by the percentage of matching trials.

To generate a chance-level of brain-behavior correspondence, we shuffled the distribution labels (actual vs. flipped) and recomputed the brain-behavior correspondence for each participant and ROI. This process was repeated for 1000 times to generate a null distribution. To compare the actual brain-behavior correspondence with the chance-level and obtain the statistical results reported in Figure 5B, we used the nonparametric permutation-based test described above to compare the actual *t*-statistic derived from comparing the actual brain-behavior correspondence for all participants with 0 to the null distribution of *t-*statistics obtained by comparing the chance-level brain-behavior correspondence for all participants with 0 in each permutation. The *p*-value at the group level was the proportion of null *t*-statistics larger than the actual *t*-statistic.

To rule out the possibility that the brain-behavior correspondence in asymmetry was driven by trials where the mean of the neural distribution was a poor predictor of behavior, we tested whether the relationship between neural and behavioral asymmetry held when the mean of the decoded distribution closely matched that of the behavioral distribution. For each ROI and participant, we selected trials where the absolute distance between the neural and behavioral means was within one standard deviation of the mean distance across all trials (see Figure S2A for the percentage of selected trials). We then repeated the brain-behavior correspondence analysis described above on these trials (Figure S2B).

### Causes of asymmetries in individual memories

To investigate the causes of asymmetries in memory, we examined the influence of task-irrelevant items and canonical motion directions (vertical, horizontal, or oblique) on decoded posteriors and the distribution of bets. The task irrelevant items we focused on were the uncued motion direction on the same trial and the cued motion direction from the previous trial. Canonical directions were defined as 90°, 135°, 180°, 225°, and 270°, and our analysis focused on the influence of these directions on targets belonging to the nearby categories of 100°, 140°, 180°, 220°, and 260°, respectively.

Neural bias was quantified by finding the likelihood over the motion direction of interest in the decoded probability distribution and subtracting the likelihood over the same position in the flipped distribution (i.e., the distribution reflected over its mean). To establish a chance-level of bias we shuffled the distribution labels (actual vs. flipped) and recomputed the bias, iterating this process 1000 times to generate a null distribution for each participant and ROI. To evaluate the statistical significance of bias and obtain the results reported in Figure 6 and Figure S3, we applied a nonparametric permutation-based test as previously described, comparing the actual *t*-statistic from the actual bias for all participants against 0, with the null distribution of *t*-statistics derived from comparing the chance-level bias for all participants with 0 across each permutation. The *p*-value at the group level was calculated as the proportion of null *t*-statistics exceeding the actual *t*-statistic.

For behavioral bias, we first centered the distribution at the first bet, as was done for the asymmetry analysis of bet distributions in ***Behavioral performance***. Then we compared the cumulative probability on the side containing the motion direction of interest with that on the opposite side. We report group-level statistics from the non-parametric paired *t*-test comparing the cumulative probability on the two sides as described above in ***Behavioral performance***.

## Supplementary Material

Supplement 1

## Data Availability

Data, experiment and analysis code, and other research materials associated with this study are publicly available on the Open Science Framework (OSF) project page https://osf.io/zc3w4/. The data comprises deidentified processed fMRI (timeseries from each voxel of each ROI).
