# Supplementary material for: Probabilistic working memory representations in human cortex guide behavior
Source: bioRxiv. 2025 Nov 17:2025.11.17.688881. Preprint. [Version 1] doi: 10.1101/2025.11.17.688881 (PMC12667906; doi:10.1101/2025.11.17.688881)
Supplement: Supplement 1 [file NIHPP2025.11.17.688881v1-supplement-1.pdf]

bioRxiv preprint doi: https://doi.org/10.1101/2025.11.17.688881; this version posted November 17, 2025. The copyright holder for this preprint (which was not certified by peer review) is the author/funder, who has granted bioRxiv a license to display the preprint in perpetuity. It is made available under aCC-BY 4.0 International license.

Extended Data (3 figures, 1 table)

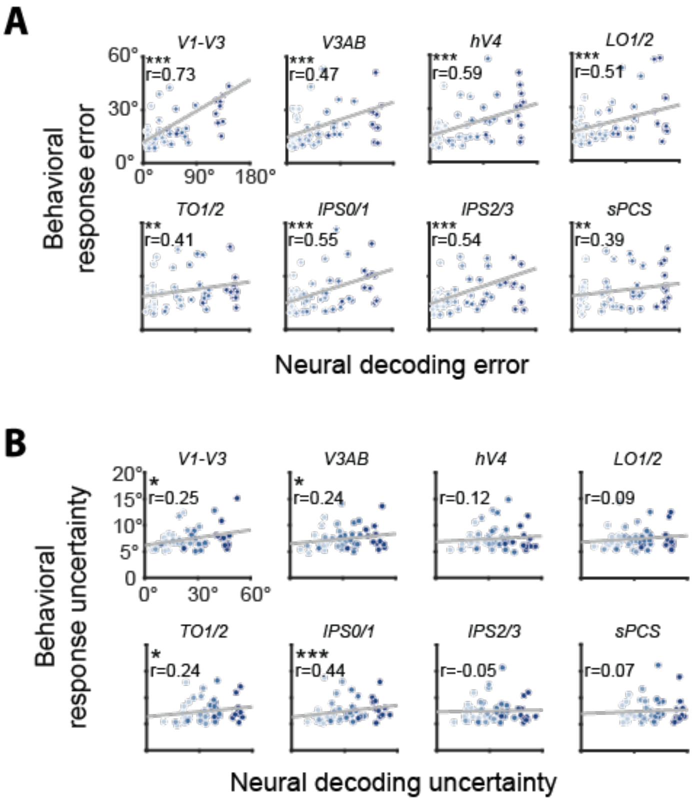

Figure S1. *Brain-behavior correspondence.* ***(A)*** *Correlation between neural decoding error and behavioral response error. The dots from lighter to darker indicate individual response error sorted and binned according to individual neural decoding error (see* ***Brain-behavior correspondence*** *for details). The gray lines are the linear fits of the dots. The stars in subplots indicate a significant correlation between the neural decoding error and behavioral response error in all ROIs (all correlations ≥ .39; ps ≤ .003, Cohen's ds ≥ 2.900).* ***(B)*** *Correlation between neural decoding uncertainty and behavioral response uncertainty. The dots from lighter to darker indicate individual response uncertainty sorted and binned according to individual neural decoding uncertainty (see* ***Brain-behavior correspondence*** *for details). The stars in subplots indicate a significant correlation between the neural decoding uncertainty and behavioral response uncertainty in V1-V3 (r = .25, p = .050, Cohen's d = 18.759), V3AB (r = .24; p = .047, Cohen's d = 2.535), TO1/2 (r = .24; p = .048, Cohen's d = .979), and IPS0/1 (r = .44; p < .001, Cohen's d = 1.967).*

bioRxiv preprint doi: https://doi.org/10.1101/2025.11.17.688881; this version posted November 17, 2025. The copyright holder for this preprint (which was not certified by peer review) is the author/funder, who has granted bioRxiv a license to display the preprint in perpetuity. It is made available under aCC-BY 4.0 International license.

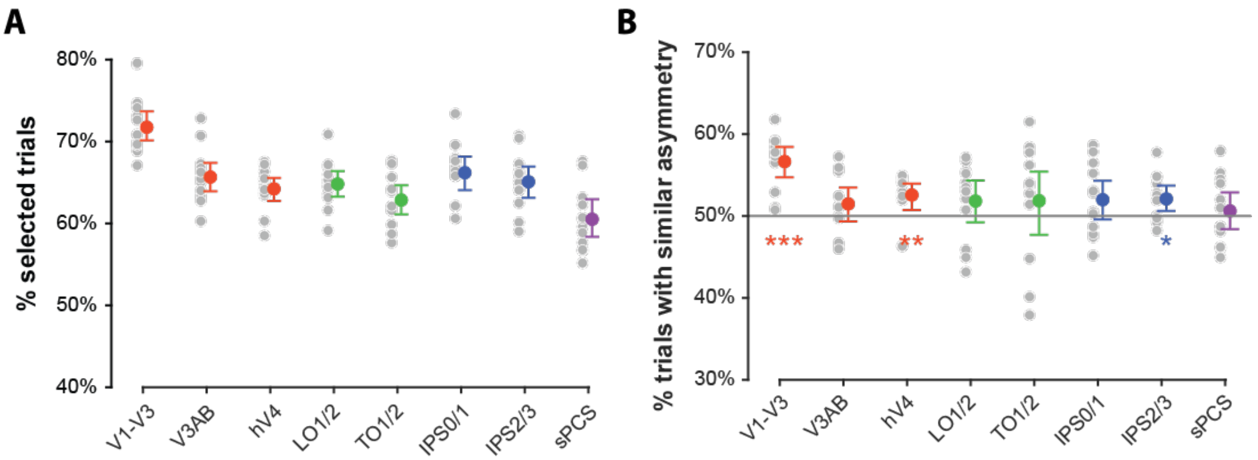

Figure S2. *Brain-behavior correspondence restricted to trials with low distance between neural and behavioral means We sought to rule out the possibility that the observed correspondence in distribution asymmetries (Figure 5) was driven by trials where the neural mean was a poor predictor of behavioral responses. Noise could theoretically shift the neural mean away from the target and preserve higher likelihoods near the target, while behavioral responses remain clustered around the target. We examined brain-behavior correspondence as in Figure 5, limiting our analysis to trials where the mean of the neural distribution was within 1 SD of the mean of the bet distribution.* ***(A)*** *Percentage of trials where the neural mean was within one SD of the behavioral mean.* ***(B)*** *Brain-behavior correspondence in these trials. The correspondence was quantified as in Figure 5B. Stars indicate significant correspondence in asymmetries in hV4 (p = .009, Cohen's d = .798), V1-V3 (p < .001, Cohen's d = 1.937), and IPS2/3 (p = .018, Cohen's d = .699).*

bioRxiv preprint doi: https://doi.org/10.1101/2025.11.17.688881; this version posted November 17, 2025. The copyright holder for this preprint (which was not certified by peer review) is the author/funder, who has granted bioRxiv a license to display the preprint in perpetuity. It is made available under aCC-BY 4.0 International license.

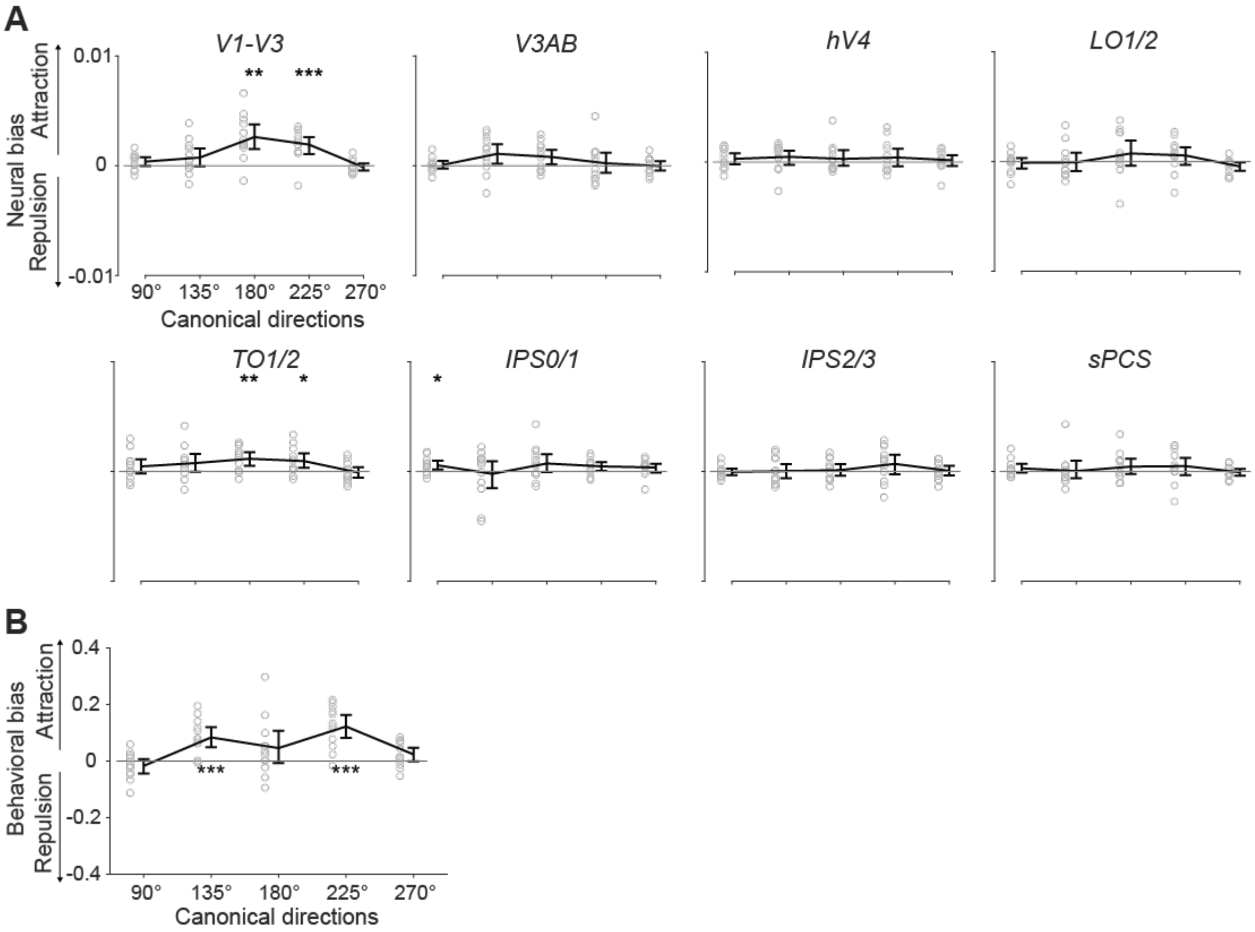

Figure S3. *Asymmetry caused by categorial bias. Trials were grouped depending on which canonical direction was closest to the cued motion direction. Each canonical direction was labeled in polar angle space.* ***(A)*** *Neural bias, quantified by calculating the likelihood over the motion direction of the nearby canonical direction and subtracting the likelihood over the same position in the neural distribution reflected about its mean. Stars indicate significant attractive biases towards canonical directions in V1-V3, TO1/2, and IPS0/1. (B) Behavioral bias, quantified by subtracting the cumulative probability at the side towards the nearby canonical direction from the side away from the nearby canonical direction, relative to the first response. Stars indicate significant attractive biases towards canonical directions in behavioral distributions.*

bioRxiv preprint doi: https://doi.org/10.1101/2025.11.17.688881; this version posted November 17, 2025. The copyright holder for this preprint (which was not certified by peer review) is the author/funder, who has granted bioRxiv a license to display the preprint in perpetuity. It is made available under aCC-BY 4.0 International license.

Table S1. Circular correlation between the decoded and target motion direction. For each subject and ROI, we report the uncorrected *p-value* obtained by comparing the actual circular correlation with a null distribution by a permutation procedure (see Methods). A *p-value* less than 0.05 (highlighted in red) indicates a significant circular correlation between decoded and target motion direction.

	**V1-V3**	**V3AB**	**hV4**	**LO1/2**	**TO1/2**	**IPS0/1**	**IPS2/3**	**sPCS**
**S1**	**0.43 (p=0.000)**	**0.23 (p=0.000)**	**0.12 (p=0.046)**	0.11 (p=0.060)	0.10 (p=0.051)	**0.49 (p=0.000)**	**0.39 (p=0.000)**	0.09 (p=0.069)
**S2**	**0.26 (p=0.000)**	**0.11 (p=0.040)**	0.09 (p=0.068)	0.01 (p=0.449)	0.08 (p=0.097)	**0.15 (p=0.005)**	**0.21 (p=0.001)**	-0.02 (p=0.638)
**S3**	**0.40 (p=0.000)**	**0.24 (p=0.001)**	**0.15 (p=0.019)**	0.12 (p=0.074)	-0.12 (p=0.943)	**0.29 (p=0.000)**	**0.31 (p=0.000)**	-0.06 (p=0.782)
**S4**	**0.51 (p=0.000)**	**0.24 (p=0.000)**	**0.20 (p=0.001)**	**0.29 (p=0.000)**	**0.24 (p=0.000)**	**0.24 (p=0.000)**	**0.19 (p=0.001)**	0.05 (p=0.219)
**S5**	**0.41 (p=0.000)**	**0.18 (p=0.000)**	**0.12 (p=0.042)**	**0.23 (p=0.000)**	**0.18 (p=0.000)**	**0.20 (p=0.001)**	**0.32 (p=0.000)**	**0.16 (p=0.008)**
**S6**	**0.28 (p=0.000)**	0.05 (p=0.264)	0.01 (p=0.496)	0.03 (p=0.356)	0.01 (p=0.451)	-0.10 (p=0.901)	0.12 (p=0.054)	0.12 (p=0.065)
**S7**	**0.17 (p=0.008)**	-0.01 (p=0.557)	0.05 (p=0.218)	0.05 (p=0.218)	**0.11 (p=0.048)**	0.00 (p=0.478)	0.11 (p=0.051)	0.07 (p=0.135)
**S8**	**0.46 (p=0.000)**	**0.44 (p=0.000)**	**0.19 (p=0.000)**	**0.35 (p=0.000)**	**0.21 (p=0.000)**	**0.23 (p=0.000)**	**0.28 (p=0.000)**	0.07 (p=0.138)
**S9**	**0.36 (p=0.000)**	**0.11 (p=0.047)**	**0.18 (p=0.001)**	**0.38 (p=0.000)**	0.03 (p=0.296)	0.08 (p=0.097)	**0.18 (p=0.003)**	-0.08 (p=0.893)
**S10**	**0.38 (p=0.000)**	**0.22 (p=0.000)**	0.11 (p=0.051)	**0.17 (p=0.002)**	0.03 (p=0.352)	**0.18 (p=0.001)**	0.10 (p=0.071)	-0.06 (p=0.821)
**S11**	**0.34 (p=0.000)**	**0.19 (p=0.002)**	0.08 (p=0.119)	0.09 (p=0.101)	-0.05 (p=0.780)	**0.21 (p=0.003)**	-0.06 (p=0.834)	-0.01 (p=0.550)
**S12**	**0.79 (p=0.000)**	**0.81 (p=0.000)**	**0.73 (p=0.000)**	**0.73 (p=0.000)**	**0.56 (p=0.000)**	**0.62 (p=0.000)**	**0.65 (p=0.000)**	**0.25 (p=0.000)**
